# Role of Hypoxia in Mesenchymal Stem Cells from Dental Pulp: Influence, Mechanism and Application

**DOI:** 10.1007/s12013-024-01274-0

**Published:** 2024-05-07

**Authors:** Muyuan Ma

**Affiliations:** School of Medicine, South China University of Technology, Guangzhou, China

**Keywords:** Dental pulp stem cells, Stem cells from human deciduous exfoliated teeth, Hypoxia, Secretome, Regenerative medicine

## Abstract

Mesenchymal stem cells (MSCs) from dental pulp (DP-MSCs), which include dental pulp stem cells (DPSCs) isolated from permanent teeth and stem cells from human exfoliated deciduous teeth (SHED), have emerged as highly promising cell sources for tissue regeneration, due to their high proliferative rate, multi-lineage differentiation capability and non-invasive accessibility. DP-MSCs also exert extensive paracrine effects through the release of extracellular vesicles (EVs) and multiple trophic factors. To be noted, the microenvironment, commonly referred to as the stem cell niche, plays a crucial role in shaping the functionality and therapeutic effects of DP-MSCs, within which hypoxia has garnered considerable attention. Extensive research has demonstrated that hypoxic conditions profoundly impact DP-MSCs. Specifically, hypoxia promotes DP-MSC proliferation, survival, stemness, migration, and pro-angiogenic potential while modulating their multi-lineage differentiation capacity. Furthermore, hypoxia stimulates the paracrine activities of DP-MSCs, leading to an increased production of EVs and soluble factors. Considering these findings, hypoxia preconditioning has emerged as a promising approach to enhance the therapeutic potential of DP-MSCs. In this comprehensive review, we provide a systematic overview of the influence of hypoxia on DP-MSCs, shedding light on the underlying mechanisms involved. Moreover, we also discuss the potential applications of hypoxia-preconditioned DP-MSCs or their secretome in tissue regeneration. Additionally, we delve into the methodologies employed to simulate hypoxic environments. This review aims to promote a comprehensive and systematic understanding of the hypoxia-induced effects on DP-MSCs and facilitate the refinement of regenerative therapeutic strategies based on DP-MSCs.

**Graphical Abstract:**

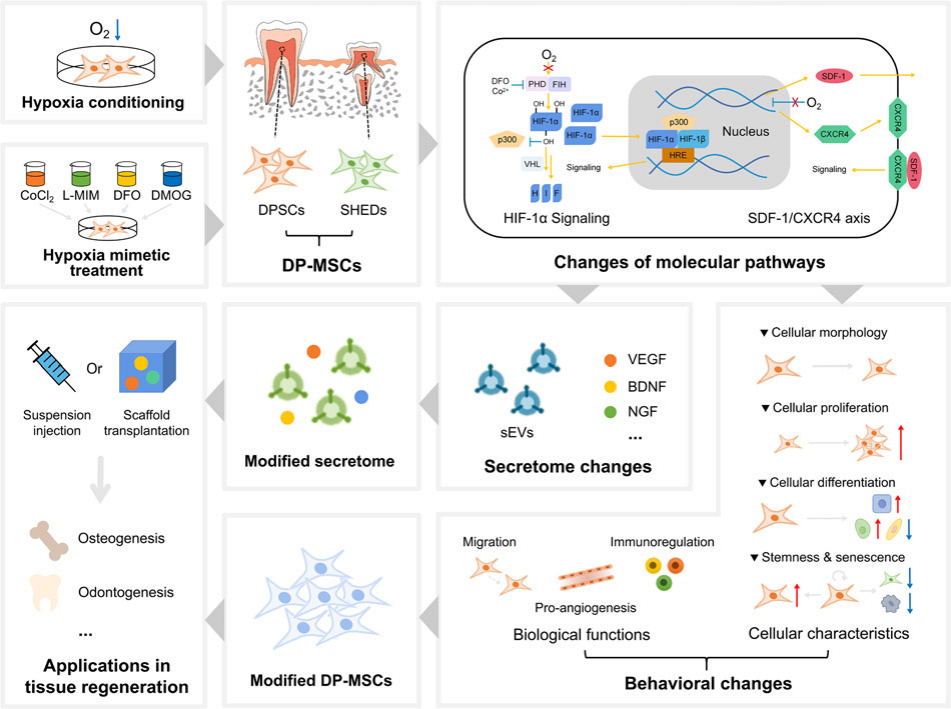

## Introduction

**Fig. 1:**
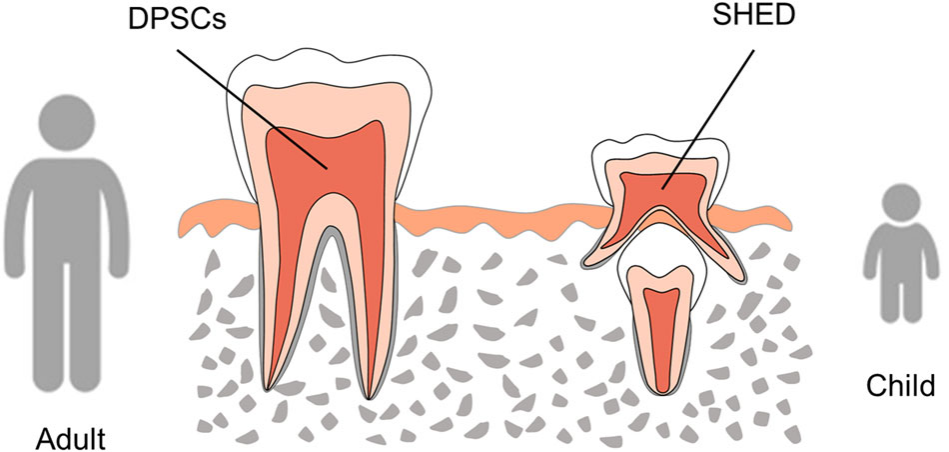
Scheme of mesenchymal stem cells (MSCs) from dental pulp (DP-MSCs). DP-MSCs consist of dental pulp stem cells (DPSCs) from adult individuals and stem cells from human exfoliated deciduous teeth (SHED) of children

Mesenchymal stem cells (MSCs) are a class of postnatal pluripotent stem cells with the potential of self-renewal, multidirectional differentiation and immune regulation, which are favorable cell sources for tissue engineering and regenerative medicine [1]. MSCs can be isolated from various tissues, including bone marrow, adipose tissue, umbilical cord, peripheral blood, synovium or dental tissue. In specific, since the first report of MSCs from dental pulp (DP-MSCs) by Gronthos et al. in [2], DP-MSCs have drawn increasing attention. DP-MSCs are ectoderm-derived stem cells with neural crest origin, which can be divided into two types, dental pulp stem cells (DPSCs) from adult individuals and stem cells from human exfoliated deciduous teeth (SHED) (Fig. 1) [3]. Both possess high proliferative rate and multi-lineage differentiation potential into odontoblasts, osteoblasts, chondrocytes, adipocytes, myoblasts, fibroblasts, neural cells, etc [4–9]. In addition, the isolation of DP-MSCs is relatively non-invasive since they are extracted from the pulp of discarded deciduous teeth in children or adult third molar after routine surgical extraction, raising rare ethical concerns [10]. Moreover, while many kinds of MSCs isolated from elderly individuals exhibit reduced biological activity with limited therapeutic potential [11], DP-MSCs isolated from elderly individuals has been demonstrated to maintain high proliferation rate and multipotency with the potential to form dentin-like matrix in vivo [12]. Thus, DP-MSCs have been growingly recognized as promising candidates for cell therapy and regenerative medicine, which have been extensively studied [13, 14].

The functionality and therapeutic effects of MSCs are often influenced by their microenvironment, also known as the stem cell niche, which includes the extracellular matrix (ECM), soluble factors such as growth factors and cytokines, neighboring cells, and physical properties such as mechanical forces and oxygen levels [15, 16]. These elements collectively create a dynamic environment that influences MSC adhesion, migration, proliferation, differentiation, and survival [15, 16]. One important factor that significantly impact MSC function is hypoxia, a state of low availability of oxygen, with both positive and negative effects [17]. Notably, since the dental pulp is surrounded by dense hard dentin tissue and receives oxygen only through the vasculature in root canals, the oxygen tension within the DP-MSC microenvironment is relatively lower than the air and the cell culture conditions [18, 19]. Additionally, hypoxia preconditioning has been proposed as an engineering approach to optimize the therapeutic potential of MSCs [20]. Thus, understanding and manipulating the hypoxic microenvironment are essential for studying the cellular biology and therapeutic application of DP-MSCs. Here, we summarize the state-of-the-art knowledge regarding the effects of hypoxia on DP-MSCs as well as the underlying mechanisms. We also highlight the applications of hypoxia-preconditioned DP-MSCs or their secretome for disease treatment. Moreover, the approaches for simulating hypoxic conditions are discussed so as to promote the optimization of DP-MSC-based regenerative therapeutic strategies.

## Behavioral Changes of DP-MSCs Induced by Hypoxia

**Fig. 2:**
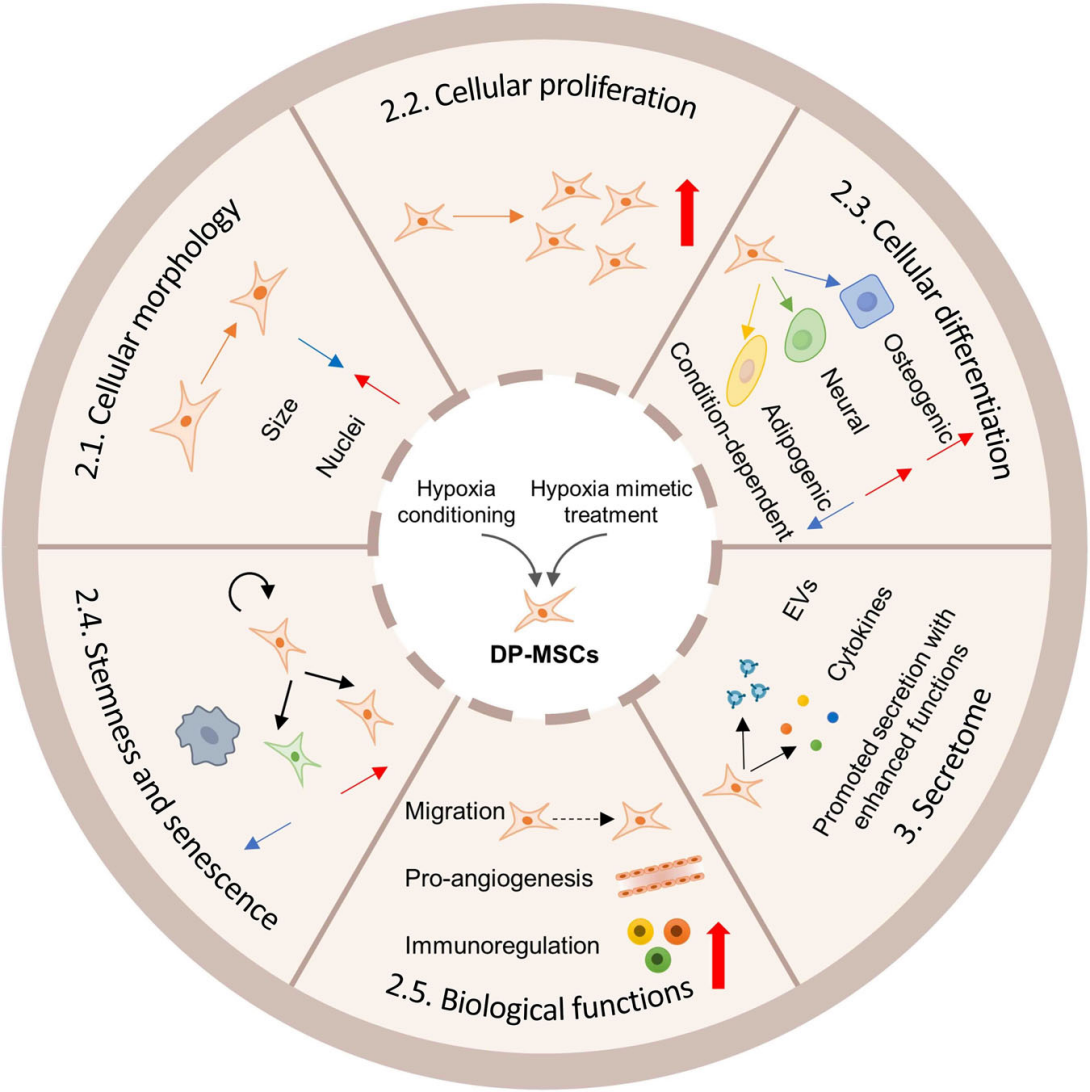
Effects of hypoxia on mesenchymal stem cells (MSCs) from dental pulp (DP-MSCs). Hypoxia is an important factor that significantly influences DP-MSCs in various aspects, including the cellular morphology, proliferation, differentiation, stemness and senescence, biological functions as well as the secretome

Considering that the oxygen tension in rat dental pulp tissue was 23.2 mmHg (approximately 3% O_2_) [18], lower than that in cell culture conditions which usually maintain 20% O_2_, a variety of studies have explored the effects of hypoxia on DP-MSCs, mostly using in vitro culture system. Research has demonstrated that hypoxia exerts a profound influence on the inherent characteristics and metabolic processes of DP-MSCs, leading to extensive alterations in their behavior, characteristics and functions (Fig. 2) [21, 22]. In practice, hypoxic conditions elicit diverse responses but generally promote the survival and functions of DP-MSCs through activation of various signaling pathways. The specific outcomes depend on various factors, such as oxygen tension variations, the origin of the pulp tissue, initial cell population, duration of culturing, and the specific culturing conditions.

### Cellular Morphology

It has been reported that DPSCs expanded in hypoxic (5%, and 3% O_2_) cultures were smaller in size compared to DPSCs in normoxic (20% O_2_) condition [23]. Also, DPSCs cultured under 3% O_2_ condition exhibited statistically larger nuclei than those cultured in 20% O_2_ condition, which may be related to higher self-renewal capacity and better differentiation potential [23]. To be noted, the changes of DPSCs morphology may become evident after long-term passages. While at early passages, cells under normoxia and hypoxia conditions exhibited similar small, spindle-shaped morphologies [23, 24], a noticeable divergence in cell morphology emerged as further passages were conducted, between normoxia and hypoxia [23].

### Cellular Proliferation

It has been demonstrated that hypoxia within a certain range has a promoting effect on DP-MSC proliferation. Iida *et al*. cultured DPSCs in 1%, 3%, 10%, and 21% O_2_ conditions and found the highest proliferation rate of DPSCs cultured in 3% O_2_ condition [12]. Similarly, DPSCs exhibited a significantly higher proliferation rate in 3% O_2_ condition compared to that in 2% O_2_ or normoxia conditions [25, 26]. Also, 5% O_2_ condition significantly enhanced the proliferation rate of DPSCs after 5 or more days of incubation, probably through the upregulation of S-phase cells [23, 24]. In addition, hypoxia treatment of DPSCs by 5% O_2_ demonstrated higher proliferation rate compared with DPSCs mobilized by granulocyte colony-stimulating factor (G-CSF) [27]. Moreover, SHED displayed even higher proliferation in terms of cell yield and a reduced doubling time under hypoxia compared to DPSCs, while preserving their phenotypic expression and differentiation potential [28]. Nevertheless, it has been demonstrated that dental pulp cells displayed a decreased proliferation rate after exposure to 2% O_2_ and 2% serum that mimics ischemic and hypoxia culture conditions [29]. This may be attributed to the activation of side population cells [29]. In another research, 5% O_2_ condition significantly compromised the growth of DPSCs, which might be related with the specific experimental settings [30].

### Cellular Differentiation

Many studies have explored the effects of hypoxia on the osteogenic differentiation of DP-MSCs. As demonstrated by Sakdee et al, hypoxia has been shown to enhance the osteogenic potential of DPSCs by promoting the upregulation of osteoprogenitor marker STRO-1 and the downregulation of primitive stem cell marker CD133 under 3% O_2_ condition [25]. Additionally, 5% O_2_ condition increased the osteogenic differentiation potential of DPSCs, as evidenced by an increased area with positive Alizarin Red S staining [24]. There is also direct evidence that 3% O_2_ condition significantly induced osteogenic differentiation of DPSCs, with significant increase in the expression of osteogenic genes BMP-2 and OCN, as well as early osteoblastic genes Runx2 and Sp-7 [31]. Notably, research has demonstrated that DPSCs cultured under 3D conditions showed elevated mRNA expression of Runx2 and Col1A1 with enhanced osteogenesis in 2% O_2_ compared to those in 18% O_2_, while more prominent osteogenesis was observed in 18% O_2_ when cultured under 2D conditions [32]. Also, hypoxic cultures of DPSCs from older patients’ teeth with inflammation have been found to suppress their osteo/odontogenic differentiation potential [12]. Moreover, studies have demonstrated that hypoxia and TNF-α have an accumulative effect in suppressing the osteogenic differentiation of DPSCs, leading to reduced expression levels of osteogenesis-associated genes such as ALP, Runx2, and OCN under osteogenic conditions, as well as decreased formation of mineral nodules as indicated by Alizarin Red S staining [30, 33]. Furthermore, long-term hypoxia culture has been reported to suppress the expression of osteogenic markers ALP and Runx2, thus inhibiting induced osteogenic differentiation [34]. Overall, the effects of hypoxia on DPSCs are complex, varying depending on multiple factors, such as cell origin, oxygen tension, incubation time, and other culturing conditions, thus emphasizing the need for further investigation and standardization. Hypoxia has also been reported to affect the differentiation capacities of DP-MSCs into other lineages. For example, hypoxic condition of 1% O_2_ was found to promote the differentiation of DPSCs into neuron-like cells with upregulation of neuronal markers nestin, β3-Tubulin, NFH, and GAP-43 [35]. Moreover, Meng et al. reported that DPSCs cultured under hypoxic condition (5% O_2_) for 14 days exhibited significant decrease in adipogenic potential, as evidenced by the suppressed expression of adipogenic markers PPAR-γ and FABP4 [34].

### Stemness and Senescence

It has been known that long-term normoxic culture induces MSC senescence and diminishes their stemness [36], which can be mitigated by hypoxia. For instance, 5% O_2_ condition has been found to significantly inhibit the senescence of DPSCs, as evidenced by a reduction in the percentage of β-galactosidase positive cells and the expression of senescence-related genes, such as P53 [24, 34]. Interestingly, one study demonstrated that while long-term hypoxia suppressed DPSC senescence, short-term hypoxia did not, indicating that the effects of suppressed senescence in 5% O_2_ condition are time-dependent [34]. In fact, the senescence-inhibiting effect of hypoxia on DPSCs during long-term culture was further confirmed by a study showing that DPSCs cultured under 3% O_2_ condition achieved 25 passages before exhaustion, compared to only 15 passages under ambient oxygen tension, and had significantly lower expression of the senescent marker β-galactosidase [37].

Moreover, hypoxia exerts promotive effects on the stemness of DP-MSCs. For example, 5% O_2_ condition induced higher expression of stem cell markers CXCR4 and G-CSFR than normoxia [23]. Additionally, Meng et al. have shown that hypoxia upregulated pluripotent genes such as OCT4, NANOG, and SOX2 in DPSCs [34]. During long term culture, the mRNA expression levels of stem cell markers SOX2, OCT4, KLF4, C-MYC and TET1 was found higher in 3% O_2_-conditioned DPSCs [37]. Expression of stemness markers STRO-1 and OCT4 has also been shown to increase in a passage-dependent manner under 5% O_2_ condition [34]. Furthermore, SHED pluripotent genes have also been found to be significantly upregulated after exposure to 3% O_2_ for 24 h and 7 days [38].

### Migration, Pro-angiogenic Potential and Immunosuppressive Function

In addition to the aforementioned effects, hypoxia has been found to impact several other functions of DP-MSCs. One such function is the migration capacity of DP-MSCs, which can be facilitated under hypoxic conditions. DPSCs cultured in 5% O_2_ condition have been observed to migrate at a higher rate towards G-CSF compared to DPSCs cultured in normal oxygen conditions [23, 27]. Similarly, both SHED and DPSCs have shown an increased rate of cell migration in 2.3% O_2_ condition, as measured by the wound healing assay, with SHED exhibiting a significantly greater effect [28]. In addition, as an important function contributing to tissue regeneration, the pro-angiogenic potential of DP-MSCs were found to be significantly enhanced by hypoxia mainly through promoting the expression of various angiogenic factors, such as hypoxia-inducible factor alpha (HIF-1α) and vascular endothelial growth factor A (VEGFA) [23, 31, 39–42], which will be further elaborated on later in this review. Furthermore, hypoxia has been found to enhance the immunosuppressive properties of DPSCs. DPSCs cultured under 5% O_2_ condition showed significantly lower expression of MHC-II, while cultures under 3% and 5% O_2_ conditions exhibited higher expression of indoleamine 2,3-dioxygenase (IDO) [23]. Additionally, the expression of the immunomodulatory gene PTGE was significantly upregulated in DPSCs conditioned in 5% O_2_, compared to G-CSF-induced mobilized DPSCs [27].

These findings suggest that hypoxia is an important factor that significantly influences the behavior, characteristics and functions of DP-MSCs in various aspects (Fig. 2). Further understanding these effects is crucial for harnessing the therapeutic potential of these cells in various regenerative medicine applications.

## Secretome Changes of DP-MSCs Induced by Hypoxia

DP-MSCs exerts extensive paracrine effects through their secretome, the sum of factors released by cells which mainly includes extracellular vesicles (EVs) and trophic factors. The synthesis and secretion of these factors are modulated by the microenvironment and the culture conditions. It has been shown that hypoxia is able to stimulate the paracrine activities of DP-MSCs and increases the production of both EVs and soluble factors (Fig. 2). Accordingly, the secretome from hypoxia-preconditioned DP-MSCs plays a crucial role in mediating the regulatory and therapeutic effects of DP-MSCs.

### EVs

EVs are nanoscale substructures that contain a diverse array of biomolecules and are secreted by cells, serving as essential mediators for intercellular communication. There are various subtypes of EVs, such as endosome-origin (exosomes), plasma membrane-derived (ectosomes) and apoptotic bodies derived from the apoptotic process [43]. Small extracellular vesicles (sEVs), or the so-called “exosomes”, are 40–100 nm spherical lipid bilayer vesicles considered to play a crucial role in various biological processes and have been investigated in many studies [44]. Since EVs usually recapitulate the biological characteristics of their parent cells and exhibit similar effects [45], pretreating MSCs under different conditions is a feasible method to alter the quantity, contents and properties of their EVs [46], among which hypoxia preconditioning is a widely studied strategy. It has been demonstrated that hypoxia-treated SHED secreted higher concentration of sEVs [42, 47], which might be attributed to the upregulation of Rab27a protein [42]. Promoted release of sEVs with increased protein contents was also reported in DPSCs under hypoxia [48]. Moreover, Li et al. found that the proteomics of sEVs derived from hypoxia-preconditioned DPSCs were significantly different from those from normoxic DPSCs [49]. Accordingly, hypoxia preconditioning further enhances the regulatory effects of those EVs.

Emerging evidence suggests that EVs derived from DP-MSCs cultured in hypoxic conditions possess augmented pro-angiogenic potential, aligning with the enhanced pro-angiogenic capacity observed in hypoxic DP-MSCs. For instance, Liu et al. conducted a study demonstrating that sEVs obtained from SHED preconditioned in 2% O_2_ significantly promoted the growth, migration, and tube formation of endothelial cells in vitro [42]. Additionally, the in vivo matrigel plug assays revealed a notable increase in micro-vessel formation surrounding the plugs containing hypoxic sEVs, characterized by elevated expression of VEGF and a greater number of lumenal structures stained by CD31 [42]. The researchers further elucidated that the pro-angiogenic effects of sEVs from hypoxic SHED could be attributed to the delivery of let-7f-5p and miR-210-3p to endothelial cells [42]. Another recent study demonstrated that sEVs derived from hypoxic SHED promoted the migration, expression of angiogenesis markers, and tube formation, as well as increased the number of junctions and overall vessel length in human umbilical vein endothelial cells (HUVECs), primarily through upregulation of the VEGF signaling pathway [47]. Moreover, it has also been demonstrated that hypoxia enhanced the pro-angiogenic potential of DPSC-derived sEVs, thereby facilitating HUVEC proliferation, migration, tube formation and VEGFA expression in vitro, which involves lysyl oxidase-like 2 (LOXL2) [49].

It has also been demonstrated that sEVs from hypoxic DP-MSCs have enhanced pro-osteogenic capacity. For example, sEVs from hypoxic SHED exhibited superior potential in promoting the osteogenic capacity of bone marrow MSCs, with higher ALP production, Ca^2+^ content and OCN expression [47]. In addition, hypoxic DPSC-derived sEVs demonstrated higher efficacy in promoting M2 macrophage polarization and suppressing osteoclast formation, thereby alleviating LPS-induced inflammatory calvarial bone loss to a greater extent compared with normoxic DPSC-derived sEVs, which indicates stronger immunosuppressive function [48]. The underlying mechanism involves the significant alteration of miRNA profiles in DPSC-sEVs due to hypoxia preconditioning. Particularly, hypoxic DPSC-derived sEVs are enriched with miR-210-3p, which exerted dual effects of inducing M2 macrophage generation and inhibiting osteoclastogenesis [48]. Furthermore, EVs from hypoxia-preconditioned DPSCs has been reported to delay the premature senescence of MSCs as well as restore MSC stemness and change their metabolism. In specific, Mas-Bargues et al. examined the effects of EVs derived from DPSCs cultured in 3% O_2_ condition on prematurely senescent DPSCs cultured under commonly used oxygen culture conditions (21% O_2_) [50]. Results showed that the senescent DPSCs treated with EVs exhibited reduced SA-β-galactosidase activity levels and increased expression of pluripotency factors (OCT4, SOX2, KLF4, and cMYC), accompanied by an increase in glycolysis and a decrease in oxidative phosphorylation (OXPHOS) [50]. Molecularly, the EVs triggered HIF-1α upregulation in target cells *via* inducing the upregulation of miR-302b, which mediated the above effects [50].

### Soluble Factors

DP-MSCs also exert extensive paracrine effects through release of various trophic factors, such as angiogenic factors and neurotrophic factors, the secretion of which are influenced by hypoxia as well. Upregulation of angiogenic factors, VEGFA and Ang-2, were found in hypoxia-conditioned dental pulp cells [40]. The expression of VEGF was also upregulated in DPSCs under 1% and 3% O_2_ conditions [31, 39, 41]. Similarly, exposure to 2% O_2_ increased VEGF expression in SHED [42]. In addition, hypoxic DPSCs cultured in 5% O_2_ condition exhibited enhanced expression of angiogenic factor VEGF, as well as neurotrophic factors BDNF and NGF [23]. The secretome collected from these cultures demonstrates higher stimulatory effects on the proliferation and migration of NIH3T3 cells, as well as neuronal differentiation of SH-SY5Y cells [23]. Further research has found increased expression of angiogenic factor GM-CSF and neurotrophic factors BDNF and NGF in DPSCs cultured under 5% O_2_ condition compared to G-CSF-induced mobilized DPSCs, the conditioned medium from which promoted greater neurite outgrowth [27]. Additionally, a multi-domain peptide hydrogel system was used to culture SHED within 3D hydrogel constructs and challenge them with hypoxic stresses via addition of H_2_O_2_ [51]. As a result, the factors obtained from lyophilized SHED cell constructs have the potential to transform lipopolysaccharide (LPS)-primed macrophages, shifting their phenotype from proinflammatory to pro-resolving [51]. Moreover, one study investigated the impact of hypoxia on DPSCs cultured in 3D *via* proteomic sequencing [41]. A total of 2115 proteins were identified, with 57 exhibiting significant differences after hypoxic preconditioning (30 up-regulated, 27 down-regulated) [41]. Bioinformatic analysis showed that the majority of up-regulated proteins are involved in angiogenesis, protein binding and transport, regulation of response to stimulus, metabolic processes, and immune response [41]. The increase of IL-6 and the decrease of TGF-1β protein expression was further confirmed by ELISA under hypoxic conditions [41].

Overall, hypoxic preconditioning has been reported to not only affect the behavior of DP-MSCs but also affect their secretome (Fig. 2). Further elucidating the involved molecular mechanism would help understand the underlying specific pathways and regulatory factors, which will promote the development of optimized hypoxia preconditioning protocols and the application of modified DP-MSCs and their secretome.

## Molecular Pathways Engaged in Changes of DP-MSCs Induced by Hypoxia

Extensive research has demonstrated that hypoxia conditions induce various changes in the molecular signaling pathways of DP-MSCs, which has been confirmed through transcriptomic and proteomic analyses. As state above, a comprehensive proteomic analysis of 3D cultured DPSCs involving 2115 proteins showed 57 proteins with significantly altered expression after hypoxic preconditioning [41]. Similarly, microarray analysis revealed the regulation of 60 mRNAs, 47 lncRNAs, and 14 miRNAs in hypoxic DPSCs [26]. Another proteomic analysis of hypoxic SHED identified 164 upregulated and 103 downregulated proteins [47]. The interplay between these molecules leads to fine-tuning changes in the signaling pathways, which mediate the multiple functional changes in DP-MSCs in response to hypoxia.

### HIF-1α Signaling

HIF is a family of transcription factors consists o a heterodimer of a constitutively expressed subunit, HIF-β, and an oxygen-regulated subunit, HIF-α [52]. In particular, the regulation of HIF in response to hypoxia relies on activity of prolyl-4-hydroxilases (PHDs) and factors inhibiting HIF-α (FIHs). While the HIF-α subunits (HIF1-α, HIF2-α, or HIF3-α) are hydroxylated and remain inactive under normoxic conditions, the HIF-α hydroxylation is inhibited under hypoxic conditions. Consequently, the HIF-α subunit is translocated to the nucleus where it dimerizes with the HIF-β subunit and activates the expression of various genes involved in adaptive responses to oxygen deprivation [53, 54]. This includes genes related to angiogenesis, erythropoiesis, glucose metabolism, and cell survival. It has been extensively shown that the expression of HIF-1α is significantly upregulated in both DPSCs and SHED under hypoxia condition, and plays a critical role in mediating the downstream effects [24, 31, 39, 41, 42, 49].

VEGF proteins is recognized as a major target gene of HIF1-α. Under hypoxic conditions, HIF1-α activates VEGF transcription to increase the production and secretion of VEGF proteins, which plays a crucial role in promoting angiogenesis [55–57]. As previously mentioned, the expression of VEGF in both DPSCs and SHED has been found to be enhanced under different concentrations of hypoxia ranging from 1% to 5% O_2_ [23, 31, 39, 42]. In the case of DPSCs, it has been observed that the inhibition of HIF-1α using the YC-1 inhibitor partially suppressed the expression of VEGF under hypoxic conditions [39]. These findings strongly suggest that HIF-1α activates VEGF expression in DPSCs in response to hypoxia. Moreover, it has been demonstrated that the increased secretion of VEGF through activation of HIF-1α aligns with the enhanced pro-angiogenic properties of SHEDs, which are crucial for maintaining long-term cell survival after transplantation [58]. Additionally, the VEGF signaling pathway has been identified as a key mediator of the pro-angiogenic potential of sEVs secreted by hypoxic DPSCs. This is supported by the observation that HUVECs treated with these sEVs exhibited increased mRNA transcription and protein expression of VEGFA [49].

LOXL2 belongs to the lysyl oxidase family, which catalyzes the deamination of lysines and hydroxylysines, and promotes the cross-linking of elastin and collagen in the extracellular matrix. Emerging evidence suggests the existence of a regulatory loop between LOXL2 and HIF-1α, with hypoxia-induced upregulation of LOXL2 being directly mediated by HIF-1α, and LOXL2 in turn regulating the HIF-1α/VEGF signaling pathways [59–61]. It has been reported that LOXL2 silencing in DPSCs inhibited their proliferation and migration [62]. Additionally, Li et al. found that LOXL2 was upregulated in both hypoxia conditioned DPSCs and their secreted sEVs [49]. Also, these sEVs could facilitate HUVECs proliferation, migration and tube formation with upregulation of LOXL2, and rescue the inhibition of tube formation caused by LOXL2 silencing in HUVECs [49]. Their further study showed that LOXL2 silencing in hypoxic DPSC-derived sEVs partially reversed the promotion of HUVEC migration and tube formation, and inhibited the expression of angiogenesis-associated genes, indicating that LOXL2 plays an important role in mediating the angiogenic effects of hypoxic DPSC-derived sEVs [62].

ATP binding cassette, subfamily G, member 2 (ABCG2) is a well-defined efflux transporter found in a variety of tissues which plays physiologically and pharmacologically important roles. Since ABCG2 is able to extrude xenobiotic compounds out of cells, the upregulation of ABCG2 expression by HIF-1a may be of significant importance for maintaining cell survival by protecting cells from accumulation of toxic compounds under hypoxia [63–65]. ABCG2 is also an important determinant of the side population phenotype and a stem-like cell marker [66]. Notably, the expression of ABCG2 mRNA was increased and ABCG2-positive cells was observed in the odontoblastic layer under ischemic hypoxia in vivo, suggesting an enhanced capacity of endogenous dental pulp cells to excrete metabolites [67]. Analogously, the expression of ABCG2 in dental pulp cells was increased after exposure to 2% O_2_ and 2% serum, with significantly higher side population proportion [29].

Furthermore, HIF-1α has been reported to decrease endogenous ROS through trans-activating the gene encoding pyruvate dehydrogenase kinase 1 (PDK1) which inactivates the TCA cycle enzyme, pyruvate dehydrogenase (PDH) which converts pyruvate to acetyl-CoA [68]. In specific, DPSCs cultured under 3% O_2_ condition showed significantly lower levels of ROS [37]. In case of SHED, HIF-1α signaling was crucial to their survival at early post-implantation stages by maintaining ROS homeostasis and inducing OXPHOS -to-glycolysis metabolic adaptations [58].

### Stromal Cell-derived Factor 1 (SDF-1)/C-X-C Motif Chemokine Receptor 4 (CXCR4) Axis

The SDF-1/CXCR4 axis plays a critical role in stem cell biology, with SDF-1 being an important chemokine responsible for attracting stem cells expressing the CXCR4 receptor to injured tissues [69]. Thus, this axis serves as a key regulatory mechanism in tissue repair and regeneration through facilitating the homing and localization of stem cells [69]. Additionally, SDF-1, *via* activating the CXCR4 receptor, can influence stem cell proliferation, differentiation, and survival through various signaling pathways [70]. It has been reported that the SDF-1/CXCR4 axis plays an important role in the hypoxia preconditioning effects of DP-MSCs.

Ahmed et al. demonstrated that DPSCs cultured in 3% O_2_ condition expressed significantly higher levels of CXCR4 compared to those cultured in 20% O_2_ condition [23]. Similarly, DPSCs cultured in 5% O_2_ condition showed higher expression of CXCR-4 and increased migration rates compared to DPSCs mobilized by G-CSF induction [27]. Wu et al. found that the expression levels of CXCR4 in DPSCs was gradually increased under hypoxic treatment, along with enhanced migration in response to SDF-1, which could be blocked by the application of the CXCR4 antagonist AMD3100 [31]. Further in vivo transplantation of hypoxia-preconditioned DPSCs through intravenous injection into apical periodontitis bone destruction model exhibited upregulated DPSC recruitment into lesion aera [31]. Their findings suggest that the interaction between SDF-1 and CXCR4 plays a crucial role in the homing of hypoxia-preconditioned DPSCs. To be noted, there is evidence suggesting that SDF-1 expression in dental pulp cells is lower under 1% O_2_ condition compared to normoxic conditions, while the expression of CXCR4 and the cell migration ability was increased [40, 71]. Furthermore, sEVs derived from hypoxic DPSCs have been found to increase the expression of SDF-1 and CXCR4 in HUVECs, suggesting that the SDF-1/CXCR4 axis is also involved in the pro-angiogenic effects of these sEVs [49].

## Applications of Hypoxia-preconditioned DP-MSCs and their Secretome in Tissue Regeneration

Cell therapy and tissue engineering based on DP-MSCs, in which DP-MSCs or DP-MSCs combined with scaffolds are transplanted systemically or locally into the body, provides promising therapeutic paradigm for regenerative medicine, predominantly for defected dental tissues, bones and nerve system. In specific, hypoxia preconditioning has shown great promises in boosting the efficacy of DP-MSC-based therapies by promoting their expansion, engraftment, survival, and therapeutic effects. To be noted, the limited number of isolated DP-MSCs necessitates their expansion before implantation while preserving their stem cell properties, for which hypoxia preconditioning is able to enhance both the proliferation rate and stemness of DP-MSCs [27]. Additionally, hypoxia preconditioning will promote the homing of systemically infused DP-MSCs in vivo *by* improving their migration [31, 72]. Moreover, the relatively hypoxic environment at transplantation sites poses a barrier to reliable therapeutic approaches, while conventional cell culture is performed at an ambient O_2_ concentrations [73, 74]. Therefore, mimicking a hypoxic microenvironment through hypoxia preconditioning before transplantation provides a feasible strategy for enhancing their adaptability and survival (Table 1).

**Table 1:** Application of hypoxia-preconditioned DPSCs and SHED as well as their secretome for in vivo tissue regeneration

Cell type	Animal Model	Hypoxia preconditioning	Application	Effects	References
Human DPSCs	Mouse apical periodontitis bone destruction model	3% O_2_	Intravenous injection of hypoxia-primed human DPSCs	• Up-regulated DPSCs recruitment • Enhanced vascularization • Enhanced osteogenesis • Suppressed local inflammation • Enhanced bone regeneration	[31]
Mouse DPSCs	Mouse calvarial critical-sized defect model	5% O_2_	Application of dense collagen scaffold containing 5% hypoxia-primed DPSCs	• Enhanced angiogenesis • Improved bone healing	[75]
Human DPSCs	Rat in situ pulp regeneration model	2% O_2_	Application of hypoxia-primed human DPSCs/nanofibrous spongy microspheres (NF-SMS) complexes	• Promoted pulp-like tissue formation with a rich vasculature and a histological structure similar to the native pulp	[76]
SHED	Enclosed in human tooth slices and implanted subcutaneously in mice	1% O_2_	Application of hypoxia-primed SHED encapsulated in a 3D collagen matrix	• Increased vascularization	[77]
SHED	Mouse critical size calvarial defect model	1% O_2_	Application of SHED-seeded dense hydrogel scaffolds primed with hypoxia	• Increased bone formation with a more compact extracellular matrix arrangement	[78]
SHED	Injection into root canals of human tooth fragments and implanted subcutaneously in mice	HIF-1α stabilization *via* knockdown of PHD2 using lentiviral shRNA	Application of HIF-1α stabilized SHED encapsulated in PuraMatrix hydrogel	• Higher level of vascularization • Decreased DNA damage and cell apoptosis • Enhance cell survival	[79]
Rat DPSCs	Mouse LPS-induced inflammatory calvarial bone loss model	1% O_2_	Simultaneous injection of hypoxic DPSC-derived sEV with LPS	• Ameliorated LPS-induced inflammatory bone loss • Induced macrophage M2 polarization • Inhibited osteoclastogenesis	[48]
SHED	Rat 5 mm-calvarial defect model	1% O_2_	Application of loaded porous PDA modified PLGA microspheres loaded with hypoxic SHED-derived exosomes	• Remarkable vascularized new bone formation with collagen-rich extracellular matrix	[47]
Human DPSCs	Mouse distraction osteogenesis model	1% O_2_	local injection of conditioned media (CMs) collected from	• Higher X-ray density • Accelerated bone healing • Improve distraction osteogenesis procedure	[40]

It has been reported that hypoxia preconditioning promoted the recruitment of intravenously transplanted DPSCs in a mouse model of bone destruction induced by apical periodontitis, resulting in favorable restoration of alveolar bone mass in the infected periapical tissue [31]. Further mechanism analysis showed that the beneficial effects was mediated through the SDF-1/CXCR4 axis [31]. In another study, the application of dense collagen scaffold containing hypoxia-primed DPSCs by 5% O_2_ effectively repaired calvarial critical-sized defects [75]. Similarly, after injection of hypoxia-primed hDPSCs/nanofibrous spongy microspheres complexes into the cleaned pulp cavities of rabbit molars which were then subcutaneously implanted in mice, significant improvements in angiogenesis and the formation of odontoblast-like cells along the dentin-pulp interface was observed within the pulp chamber [76]. Further application into an in situ dental pulp repair model of rats exhibited the regeneration of pulp-like tissues with well-developed vascular networks and histological structure closely resembling that of the native pulp [76]. Moreover, hypoxia-primed SHED were encapsulated in hydrogels and placed into the empty pulp chamber space, which also demonstrated enhanced angiogenesis after subcutaneous implantation in vivo [77]. Consequently, the application of such tissue-engineered hydrogels seeded with hypoxia-primed SHED resulted in faster and better bone formation in a craniofacial bone repair model [78]. Furthermore, Han et al. preconditioned SHED by stabilizing HIF-1α through knockdown of PHD2, and then encapsulated HIF-1α-stabilized SHED in PuraMatrix hydrogel, which were injected into the root canals of human tooth fragments and subsequently implanted in the subcutaneous space of immunodeficient mice [79]. At 7 days post-implantation, the HIF-1α-stabilized SHED group exhibited significantly less DNA damage and higher expression of Ki67 compared to the control group, suggesting improved survival of transplanted SHED. After 28 days, remarkable formation of dental pulp-like tissue with significantly higher vascularization and accelerated odontogenic/osteogenic differentiation was observed [79].

Although DP-MSCs have exhibited remarkable tissue regeneration potential, direct application of stem cells are confronted with many obstacles. In specific, patient safety is one of the major concerns due to the potential for side effects, such as tumor formation or immune rejection following transplantation. Moreover, the therapeutic efficacy of DP-MSCs in vivo may be severely limited due to reduced survival and implantation efficiency [80]. Notably, within recent years, cell-free strategies, which use the secretome of MSCs while removing the cells themselves, have shown encouraging therapeutic potential with fewer safety considerations and simplified clinical management [51, 81]. As discussed above, the application of hypoxia treatment has been demonstrated to elicit a notable elevation in the secretion of EVs and bioactive factors from both DPSCs and SHED, which also provides a feasible method to improve their biological functions [42, 46–48].

A study conducted by Tian et al. demonstrated that hypoxia significantly promoted the release of sEV from DPSCs, which exhibited superior effectiveness in promoting M2 macrophage polarization and suppressing osteoclast formation, thereby alleviating LPS-induced inflammatory calvarial bone loss [48]. Additionally, hypoxia preconditioning has been shown by Gao et al. to enhance the secretion of exosomes from SHED, which exhibited enhanced potential in promoting angiogenesis and osteogenesis [47]. They further developed a platform technology utilizing injectable porous poly(lactide-co-glycolide) (PLGA) microspheres coated with bioinspired polydopamine (PDA) for effective delivery of hypoxic SHED-derived exosomes [47]. This technology enables sustained release kinetics of hypoxic exosomes, with high bioactivity observed for 21 days, ultimately leading to vascularized bone regeneration in a 5-mm rat calvarial defect model [47]. Moreover, Fujio et al. collected conditioned media from human DPSCs under either hypoxia or normoxia culture conditions and injected locally into mice performed with distraction osteogenesis in vivo [40]. Results showed that the hypoxic conditioned media has significantly higher bone healing capacity than normoxic conditioned media, which might be due to the enhanced angiogenic potential [40]. These findings highlight the potential of the secretome of hypoxic DP-MSCs as a promising therapeutic approach for tissue regeneration.

## Pharmacologic Interventions Simulating Hypoxic Preconditioning

Despite the favorable cellular effects of hypoxia preconditioning, the requirement for a complex hypoxic-inducing environment, such as a hypoxia chamber, makes it necessary to develop simpler and safer strategies that are more suitable for use in both laboratory and clinical settings. To address this issue, researchers have begun to explore some simpler methods for achieving cellular hypoxia preconditioning. One such method is to use chemical substances to simulate hypoxic conditions. These hypoxia mimetic agents are able to mimic the cellular response to low oxygen environments, most of which relies on the stabilization of the HIF-1α [82, 83]. This method is relatively simple and easy to perform, requiring no complex equipment, making it more easily applicable. Another simplified approach to hypoxia preconditioning is through the use of biotechnological tools, such as gene editing technology. By editing the genome of cells, it is possible to directly modify oxygen sensors or related signaling pathways within the cell, making the cell more sensitive to low oxygen environments. In this regard, silencing the expression of PHD2 to stabilize HIF-1α expression has been demonstrated to be an appealing strategy for mimicking hypoxic preconditioning, with beneficial effects on DP-MSCs [74, 79]. The exploration of simpler and safer strategies will provide potential alternatives for cellular research and clinical treatment.

Cobalt chloride (CoCl_2_) is a commonly used hypoxia-mimetic agent conferring hypoxia tolerance by stabilization of HIF-1α [84]. Laksana et al. reported that CoCl_2_ dose-dependently induced the expression of stem cell markers REX1, OCT4, SOX2, and NANOG in human dental pulp cells and increased the number of STRO-1 positive cells, while suppressing osteogenic-associated gene expression, ALP activity, and calcium deposition [85]. Notably, the inhibitory effect of CoCl_2_ on ALP activity was reversed when HIF-1α inhibitor apigenin was added [85]. Zheng et al. also found that CoCl_2_ inhibited ALP activity and suppressed the mineralization of DPSCs, with down-regulated OCN, DSPP, DMP1, and BSP protein expression, which could be rescued by miR-140-3p overexpression [86]. Furthermore, CoCl_2_ has been shown to increase the expression of stem cell markers OCT4, NANOG, SOX2, and c-Myc, and promote the migration ability of SHED, while inhibiting osteogenic differentiation with reduction in ALP activity and calcium deposition, as well as the expression of osteogenic-related genes [87]. These findings indicate that CoCl_2_ is able to improve the stemness of DP-MSCs while partially suppressing their osteogenic capacity through HIF-1α activation, supporting the use of CoCl_2_ in maintaining the undifferentiated state of DP-MSCs in the laboratory by mimicking a hypoxic environment in vitro.

L-mimosine (L-MIM), a non-protein amino acid, blocks the active site of the oxygen sensors PHD and FIH, and also chelates Fe^2+^. Both L-MIM and hypoxia increased the angiogenin protein expression in monolayer cultures of dental pulp cells, whereas only L-MIM significantly increased the angiogenin protein level in spheroid cultures [88]. Analogously, both L-MIM and hypoxia increased angiogenic factor Angptl4 production in dental pulp cells with increased expression of HIF-1α [89]. These findings indicate that treating with L-MIM provides a useful method for improving the angiogenic capacity of DP-MSCs.

Desferrioxamine (DFO) chelates Fe^2+^, and thereby inhibits PHD 3 and 1 as well as FIH-1. It has been demonstrated that 10 μM DFO enhanced the expression of HIF-1α in dental pulp cells and enhanced their proliferation, migration, and odontogenic differentiation [90]. Further study demonstrated that 10 μM DFO promoted the secretion of SDF-1α or VEGF in dental pulp cells and improved migration of them through HIF-1α, as the effects of DFO on dental pulp cells were partially reversed by HIF-1α silencing [91].

Dimethyloxalylglycine (DMOG) is a highly specific hypoxia mimetic agents that structurally mimics 2-oxoglutarate and therefore inhibits PHDs to stabilize HIF-1α [92]. In a study utilizing synthetic clay-based hydrogels to carry dental pulp cells, it was found that hydrogels loaded with DMOG can release DMOG and induce a proangiogenic response in dental pulp cells. Additionally, the VEGF produced by these cells can bind to the synthetic clay [93].

Collectively, pharmacologic interventions, such as the use of hypoxia mimetic agents, provide a more reliable and consistent approach to maintain a hypoxic environment. The concentrations of these agents can be easily adjusted and controlled, allowing for precise modulation of cellular responses. Thus, pharmacologic interventions will offer promising prospects in simulating hypoxia for various applications in regenerative medicine.

## Conclusion

DP-MSCs possess multipotential differentiation ability, superior proliferation and self-renewal potential, and low immunogenicity, highlighting their promising applications in the field of regenerative medicine. Notably, the microenvironment, particularly the level of oxygen, plays a critical role in modulating the behavior and function of DP-MSCs. Consequently, hypoxia preconditioning is being increasingly evaluated as a very attractive strategy for optimizing their functions and applications. Further exploration of the involved molecular mechanism would help understand the specific pathways and regulatory factors underlying this beneficial effect, thus leading to the development of targeted therapeutic strategies to optimize hypoxic preconditioning protocols and maximize the therapeutic potential of DP-MSCs in various clinical applications. Additionally, investigating the long-term effects and stability of the altered gene and protein expression profiles induced by hypoxia preconditioning would provide valuable insights into the durability and persistence of these enhanced regenerative properties. Moreover, exploring the interactions between hypoxia preconditioning and other influential factors, such as growth factors or scaffolds, may uncover synergistic approaches to further enhance the therapeutic outcomes of DP-MSC transplantation for tissue repair and regeneration. In summary, understanding the effects and mechanism of hypoxia modulation on DP-MSCs will promote the establishment of optimized tissue regeneration strategies based on DP-MSCs and their secretome.
